# Fatty meal sonography comparing coconut oil and chocolate bar with full-fat yoghurt as cholecystagogues for gallbladder ejection fractions

**DOI:** 10.4102/sajr.v22i1.1312

**Published:** 2018-06-18

**Authors:** Benjamin Spangenberg, Jacques Janse van Rensburg

**Affiliations:** 1 Department of Radiology, University of the Free State, South Africa; 2 Department of Clinical Imaging Sciences, Universitas Hospital, South Africa

## Abstract

**Background:**

The authors compared the effectiveness of a chocolate bar and full-fat yoghurt combination to coconut oil in determining the gallbladder ejection fraction (GBEF). The clinical motive was functional gallbladder disorder (FGD) which has the clinical picture of symptomatic gallstones but without gallstones. Functional gallbladder disorder has a decreased GBEF of less than 35%. Gallbladder ejection fraction can be calculated by ultrasound, using cholecystokinin (CCK) as a stimulant for gallbladder contraction. Cholecystokinin is not available in South Africa, and the researchers compared a 60 g Snickers chocolate bar with 200 g full-fat yoghurt, against the theoretically superior coconut oil.

**Objectives:**

To determine the efficacy of coconut oil versus chocolate bar and 200 g full-fat yoghurt combination in performing sonographic GBEFs.

**Method:**

This was a randomised clinical experiment, before and after crossover trial. The three experimental components of the study included 15 g coconut oil, 20 g coconut oil and a standard fatty meal consisting of 60 g Snickers bar and 200 g full-fat yoghurt.

**Results:**

The GBEF for the chocolate bar and yoghurt combination was the highest (62.84%). The GBEF for 20 g of coconut oil was 23.47% and for 15 g of coconut oil was 5.11%. There was a statistically significant difference between the chocolate and yoghurt combination and the 20 g coconut oil, as well as the chocolate yoghurt combination and the 15 g coconut oil, both with a *p*-value of < 0.0001. No statistically significant difference was found between the 20 g and 15 g coconut oil.

**Conclusions:**

The 60 g Snickers chocolate bar and 200 g full-fat yoghurt combination was superior to the coconut oil. The authors advocate using the chocolate and yoghurt fatty meal oral stimulant to determine GBEF.

## Introduction

Functional gallbladder disorder (FGD), also referred to as chronic acalculous cholecystitis,^1^ is a diagnosis considered in patients with biliary pain attributed to a primary gallbladder motility disturbance, in the absence of gallstones or gallbladder sludge. Research has demonstrated a prevalence of FGD as high as 8% in males and 20% in females who present with biliary-type pain and a normal abdominal ultrasound.^2,3^ A biliary-type pain is defined in the Rome IV criteria applicable to FGD (Table 1).^4^

**TABLE 1:** Rome IV diagnostic criteria for functional gallbladder disorder.

Criteria	Symptoms or findings
Diagnostic criteria for functional gallbladder disorder	Biliary pain Absence of gallstones or other structural pathology
Supportive criteria for functional gallbladder disorder	Low ejection fraction on gallbladder scintigraphy Normal liver enzymes, conjugated bilirubin and amylase/lipase
Diagnostic criteria for biliary pain	Pain located in the epigastrium and/or right upper quadrant and all of the following: Builds up to a steady level and lasting 30 min or longer Occurring at different intervals (not daily) Severe enough to interrupt daily activities or lead to an emergency department visit Not significantly (<20%) related to bowel movements Not significantly (<20%) relieved by postural change or acid suppression
Supportive criteria for biliary pain	The pain may be associated with: Nausea and vomiting Radiation to the back and/or right infra-subscapular region Waking from sleep

The aetiology of FGD is still unclear: proposed aetiologies include either a primary motility disorder of the gallbladder, or a motility disorder secondary to super saturated cholesterol because of an underlying metabolic disorder.^4,5^ Functional gallbladder disorder has also been linked to generalised gastrointestinal motility disorders with abnormal gastric emptying and abnormal colonic transit time.^6^

Patients with FGD present with a biliary-type pain; have normal liver enzymes, conjugated bilirubin and amylase/lipase levels; demonstrate no gallstones on ultrasound; and have normal gastroscopy findings.^4^ When a patient, thus, presents with a biliary-type pain that fulfils the Rome IV criteria (Table 1) for FGD, and where all other possible causes of a biliary-type pain have been excluded, the next step involves calculating the gallbladder ejection fraction (GBEF). If the GBEF is less than 35%, then the patient will benefit from a cholecystectomy.^7^ A study by Wybourn et al. showed that most patients respond to cholecystectomy with no pain recurrence.^8^

Cholecystokinin (CCK)-stimulated cholescintigraphy or sonography is routinely used to determine the ejection fraction of the gallbladder. Cholecystokinin, which is commercially available as Kinevac (sincalide for injection; Bracco Diagnostics Inc., Princeton, NJ), is unfortunately not available in South Africa. Alternatives that have been described in the literature include half and half milk,^9^ chocolate and full-fat yoghurt,^10^ corn oil^11^ and cottonseed oil.^12^ The latter two are also not readily available in South Africa. Previous studies have also used Lipomul (Lee Pharmaceuticals, South El Monte, CA), which was discontinued in 1979. Lipomul oral liquid is composed of hydrogenised stabilised soybean oil, water, sugar, propylene glycol, vitamins and minerals.

Cholecystokinin is secreted when fatty acids enter the duodenum. Fatty acids elicit a chain length and dose-dependent stimulation of CCK. In this regard, dodecanoic acid, also known as lauric acid, has proven to be the most effective, producing a fivefold increase in CCK secretion.^13^ Lauric acid comprises about half the fatty acid content found in coconut oil, laurel oil and palm kernel oil. It is also found in human breast milk, cow’s milk and goat’s milk,^14^ but otherwise it is relatively uncommon.

In this study, we compared the effectiveness of a chocolate bar and full-fat yoghurt combination to coconut oil in determining the GBEF. The study is based on the premise that lauric acid (in the form of coconut oil) is an effective and readily available cholecystogogue alternative to CCK in determining the GBEF.

## Research method and design

A randomised clinical experimental study was performed with a before and after cross over trial at the Universitas Academic Hospital. The study protocol was drafted by the researchers and approved by the Free State University Ethics committee as well as the Free State Department of Health (HREC 122/2016).

Forty volunteers were recruited for the study by personal invitation between June 2017 and December 2017. Volunteers were healthy men and women above 18 years of age. All the volunteers consented to the study.

Volunteers were excluded if they had any FGD symptoms as described in the Rome IV criteria, GBEF less than 35%^8^ and used any medication that would impair gallbladder emptying, including morphine, atropine and calcium channel blockers.^15^ Other exclusion criteria were participants with irritable bowel syndrome,^16^ individuals with diabetes, volunteers who were not smoke-free for at least 8 h prior to the ultrasound being performed^17^ and individuals with a gallbladder that could not be accurately measured in all three planes. If incidental gallstones were found, the volunteer was excluded from the study, informed of the findings and referred to a surgeon.

Three types of cholecystogogues were utilised in the study and included 15 g of coconut oil, which contains approximately 14.97 g of total fat, 20 g of coconut oil, which contains approximately 19.96 g of total fat and a standard fatty meal composed of 20 g of total fat, consisting of a chocolate bar and full-fat yoghurt.

The brands used were Lifestyle Foods Organic Cold-pressed Virgin Coconut Oil, which contains 99.8 g of total fat per 100 g (47.5 g of which is lauric acid) and 3693 kJ per 100 g. The 60 g Snickers chocolate bar was used with 200 g full-fat yoghurt as the standard fatty meal. This chocolate bar and yoghurt combination contained on average of 20 g fat, 16 g carbohydrates, 16 g protein and 1339 kJ.

There were three groups of participants with before and after crossover. The healthy volunteers were randomly divided into three groups. Group A received 15 g of coconut oil, group B received 20 g of coconut oil and group C received the standard fatty meal. By the end of the study, all three groups had received the different doses of coconut oil and standard fatty meal.

The principal researcher performed all the evaluative ultrasounds. A single ultrasound machine with a curvilinear ultrasound probe was used (Aloka ProSound Alpha5 SV, UST-9126 ultrasound probe, 2.0 MHz–6.0 MHz). Subjects reported to the ultrasound room after fasting overnight. A fasting ultrasound volume was recorded, and then one of the three stimulants was given to the participant to be consumed in 5 min. Forty minutes after ingesting of the stimulant, a follow-up ultrasound was performed to determine the gallbladder ejection volume. A previous study by Barr et al. found the time to peak ejection fraction at ultrasound was 38 ± 12 min^18^; thereafter, the participants could continue with their regular diet.

Each of the 40 participants had six ultrasounds: Fasting, and 40 min after the stimulant, using all three of the cholecystogogue stimulants. Each of the stimulants were utilised on different days.

The researcher was blinded to the group allocated to the participants.

**FIGURE 1:**
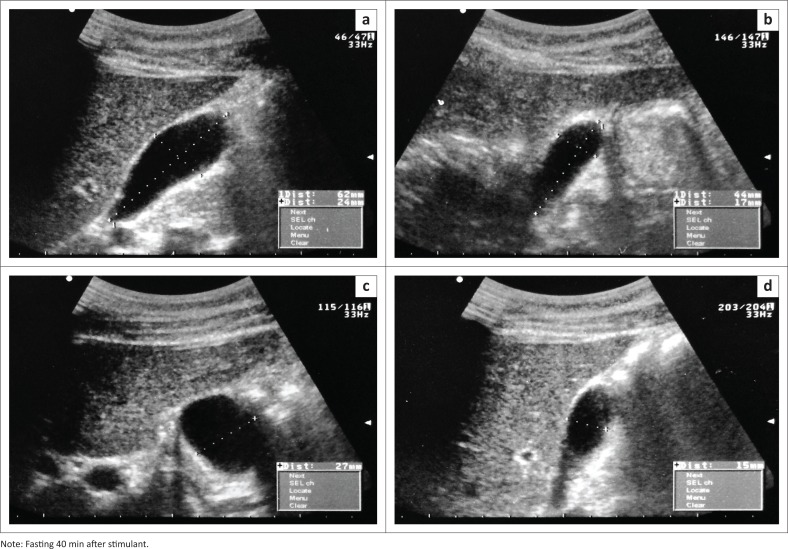
Fasting and 40 min after gallbladder stimulant, measuring the length, width and height of the gallbladder in orthogonal planes on the same participant: (a and c) fasting and (b and d) 40 min after stimulant.

Gallbladder size was measured in three orthogonal planes: length, height and width, and multiplied by 0.523 to calculate the ellipsoid volume (Figure 1). Volume = W × H × L × 0.52, where W is the gallbladder width, H = height and L = axial length.^19^ Gallbladder ejection fraction was calculated by the following formula: Fasting gallbladder volume – Residual gallbladder volume)/Fasting gallbladder volume × 100%.

The data were captured in Microsoft Excel and analysed by the Department of Biostatistics at the University of the Free State.

## Results

Forty healthy subjects were recruited. Three were excluded because of the finding of asymptomatic gallstones and were referred for surgical opinions. One participant had a gallbladder that could not be accurately measured and was excluded. One participant did not tolerate the coconut oil and did not complete the second coconut leg of the study. Three participants had a GBEF of less than 35% with the chocolate and yoghurt combination and were excluded. Thirty-two participants, thus, met the inclusion criteria (21 female, 11 male; age 20–60 years; mean age: 34 years).

The GBEF for the chocolate bar and yoghurt combination was the highest of the cholecystagogues (62.84%), comparable with previous studies.^20^ The GBEF was 23.47% for 20 g of coconut oil and 5.11% for 15 g of coconut oil.

There was a statistically significant difference between the chocolate and yoghurt combination and the 20 g coconut oil, as well as between the chocolate yoghurt and the 15 g coconut oil, both with a *p*-value of <0.0001. No statistically significant difference was found between the 20 g and 15 g coconut oil (*p*-value = 0.2380). The mean time interval between the fasting and residual volume ultrasound measurements was comparable: for the 15 g leg, 48 min; for the 20 g leg, 46 min; and for the yoghurt and chocolate combination, 46.5 min.

Upon verbal informal debriefing after the study, the chocolate bar and yoghurt combination was more palatable to the participants. Coconut oil melts at 25°C.^14^ Towards the end of the study, the samples for the study melted (owing to the South African summer temperatures) and were taken in liquid form by the participants. The participants found the liquid form easier to ingest.

## Discussion

Normally, CCK is secreted by the ‘I’ cells in the mucosa of the duodenum and jejunum. This is in response to the presence of breakdown products of fat, fatty acids and monoglycerides in the intestinal contents. This increases gallbladder contraction and causes excretion of bile from the gallbladder into the small intestine. Bile plays an important role in emulsifying fatty substances for absorption and digestion.^21^

According to McLaughlin et al., lauric acid causes a fivefold increase in CCK excretion.^13^ In that study, it was tested using STC-1, a mouse intestinal endocrine tumour cell line. In our study, about half of the fatty acids in coconut oil were lauric acid. It is possible that the human response to lauric acid is different to STC-1, just as minor differences in fatty acid secretion were noted in the same study of McLaughlin et al., in which decanoic acid was not able to secrete CCK in humans. Species variation differences may be reflected.

The coconut oil was of a much smaller volume (20 g), compared to 260 g of the chocolate yoghurt combination. The possibility that the volume has an effect is not likely. It is not duodenal and jejunal distension that causes CCK secretion but the presence of breakdown products of the fat, fatty acids and monoglycerides in the duodenum and jejunum.^21^

Matched for total fat against a well-described readily available stimulant, 200 g of full-fat yoghurt and 60 g chocolate bar combination against 20 g of coconut oil, the chocolate and yoghurt combination was the clear winner.

The aim of the study was to compare the efficacy of 15 g coconut oil and 20 g coconut oil versus the chocolate bar and 200 g full-fat yoghurt combination in performing sonographic GBEFs. The chocolate and yoghurt combination showed superior GBEF (62.84%) compared to the coconut oil. The GBEF for 20 g of coconut oil (23.47%) and the GBEF for 15 g coconut oil (5.11%) did not even pass the threshold for diagnosis of FGD (35%).

## Conclusion

The 60 g Snickers chocolate bar and 200 g full-fat yoghurt combination was found to be superior to the theoretically better coconut oil in this study. The authors advocate using the chocolate and yoghurt fatty meal oral stimulant to determine GBEF. Coconut oil is not a viable alternative in these small-volume doses, even though this was matched for total fat. The chocolate bar and yoghurt combination is also much more palatable.

### Study limitations

The number of participants, although limited, was comparable to previous studies.^20^ Because of the nature of the study, diabetics were excluded from the study. The coconut oil could not be benchmarked against intravenous CCK, as intravenous CCK is not available in South Africa.
